# Glycogene Expression Profile of Human Limbal Epithelial Cells with Distinct Clonogenic Potential

**DOI:** 10.3390/cells11091575

**Published:** 2022-05-07

**Authors:** Damien Guindolet, Ashley M. Woodward, Eric E. Gabison, Pablo Argüeso

**Affiliations:** 1 Schepens Eye Research Institute of Mass. Eye and Ear, Department of Ophthalmology, Harvard Medical School, 20 Staniford Street, Boston, MA 02114, USA; dguindolet@for.paris (D.G.); ashley.woodward@tufts.edu (A.M.W.); 2 Hôpital Fondation A. de Rothschild, Department of Ophthalmology, 25 rue Manin, 75019 Paris, France

**Keywords:** cornea, α-L-fucosidase, *GCNT4*, glycome, limbal epithelium, stem cell

## Abstract

Glycans function as valuable markers of stem cells but also regulate the ability of these cells to self-renew and differentiate. Approximately 2% of the human genome encodes for proteins that are involved in the biosynthesis and recognition of glycans. In the present study, we evaluated the expression of a small subset of glycogenes in human limbal epithelial cells with distinct clonogenic potential. Individual clones were classified as abortive or clonogenic, based on the fraction of the terminal colonies produced; clones leading exclusively to terminal colonies were referred to as abortive while those with half or fewer terminal colonies were referred to as clonogenic. An analysis of glycogene expression in clonogenic cultures revealed a high content of transcripts regulating the galactose and mannose metabolic pathways. Abortive clones were characterized by increased levels of *GCNT4* and *FUCA2*, genes that are responsible for the branching of mucin-type O-glycans and the hydrolysis of fucose residues on N-glycans, respectively. The expansion of primary cultures of human limbal epithelial cells for 10 days resulted in stratification and a concomitant increase in *MUC16*, *GCNT4* and *FUCA2* expression. These data indicate that the clonogenic potential of human limbal epithelial cells is associated with specific glycosylation pathways. Mucin-type O-glycan branching and increased fucose metabolism are linked to limbal epithelial cell differentiation.

## 1. Introduction

The glycome can be defined as all the glycan structures synthesized by an organism and is estimated to be orders of magnitude greater than the proteome. Such diversity is provided by the combined action of multiple genes encoding glycosyltransferases, glycosidases and other enzymes that synthesize and remodel glycan chains, along with the accessory enzymes involved in the synthesis and transport of nucleotide sugars [1]. The genetic material necessary to orchestrate this complex synthetic machinery has been extensively described, although less is known about how this information is translated into the synthesis of specific glycan structures [2]. Recent estimates indicate that at least 2% of the human genome is devoted to the generation of the glycome [3]. The identification of these glycogenes, as well as the resulting glycan structures, in cells and multicellular organisms, is proving critical for a better understanding of fundamental biological processes.

The glycome of any given cell lineage is highly dependent on its differentiation status and individual microenvironment. As a result, the glycome, and more specifically, the expression of specific glycans in biological tissues, has been used to assess stemness and the degree of cellular maturation. Many monoclonal antibodies, used early on in biochemical studies to characterize cell surface molecules expressed in embryonic tissues, have been found to recognize carbohydrate epitopes [4]. In stem cells, these epitopes show reactivity against different types of glycoconjugates, including glycoproteins, glycosaminoglycans and glycosphingolipids [5,6,7]. For instance, human embryonic stem cells contain complex fucosylated structures on N-glycans that change according to the differentiation stage [8]. Likewise, the induction of pluripotency in stem cells has been associated with a glycome shift leading to increased levels of high mannose-type N-glycans and α2–6 sialic acid [9]. Moreover, important to the biological function of the cell is that most of these glycan epitopes play critical roles in the regulation of cell adhesion and signaling events [10].

Stem cells can be divided into (i) pluripotent stem cells, comprising embryonic stem cells and induced pluripotent stem cells, and (ii) somatic or nonembryonic stem cells, commonly known as adult stem cells. Pluripotent stem cells can differentiate into all the cell types present in an adult organism. Somatic stem cells, on the other hand, are located in specialized niches in the adult organism and maintain tissue homeostasis by producing a progeny that replace cells that are lost as a result of tissue turnover or injury [11]. Somatic stem cells in the human corneal epithelium localize preferentially to the basal layer of the transition zone between the peripheral cornea and the anterior sclera, in an area known as the limbus [12]. These cells are slow-cycling, can self-renew and are clonogenic—the latter indicating that each cell possesses a strong potential to grow into a colony of identical cells. When cultured in the presence of lethally irradiated mouse 3T3 fibroblasts, epithelial cells from the limbus exhibit a substantial proliferative capacity with an average of 23 population doublings, whereas cells from the central cornea cannot be propagated and deteriorate in the first or second passage [13]. In the present study, we evaluated the expression profile of a small subset of glycogenes expressed by human limbal epithelial cells with distinct clonogenic properties. We find that cells with a higher clonogenic potential produce transcripts regulating galactose and mannose metabolic pathways. Moreover, we show that limbal epithelial cell differentiation and loss of clonogenic potential are associated with mucin-type O-glycan branching and an increased fucose metabolism.

## 2. Materials and Methods

### 2.1. Growth-Arrested 3T3-J2 Fibroblasts

The 3T3-J2 murine embryonic fibroblasts (Kerafast, Boston, MA, USA) were maintained in DMEM GlutaMAX™ (Thermo Fisher Scientific, Waltham, MA, USA) with 10% iron-supplemented calf serum (HyClone, Pittsburgh, PA, USA) and 1% penicillin/streptomycin in a tissue culture incubator with 5% CO_2_. The 3T3-J2 fibroblasts were mitotically inactivated by incubating the cells with 4 µg/mL of mitomycin C (MilliporeSigma, Burlington, MA, USA) for 2 h at 37 °C, and then rinsed 5 times with Dulbecco’s modified phosphate-buffered saline lacking calcium and magnesium (Thermo Fisher Scientific). The fibroblasts were harvested using TrypLE Express (Thermo Fisher Scientific) and frozen in DMEM GlutaMAX™ supplemented with 20% iron-supplemented calf serum and 10% DMSO.

### 2.2. Human Limbal Epithelial Cell Culture

Human postmortem corneoscleral tissues were generously donated by Lions VisionGift (Portland, OR, USA). The iris root and endothelium were scraped with a K-Sponge Spear (Katena, Denville, NJ, USA). The central portion of the tissue was cut with an 8-mm disposable biopsy punch (Integra Miltex, York, PA, USA) to produce corneal rims. These were subsequently incubated for 1 h at 37 °C with 2.4 IU/mL Dispase II (Thermo Fisher Scientific) in a supplemented growth medium (sGMedium) containing a 3:1 mixture of DMEM GlutaMAX™:F12 with 10% fetal bovine serum, 0.4 μg/mL hydrocortisone, 5 μg/mL insulin, 1.4 ng/mL triiodothyronine, 24 μg/mL adenine, 8.4 ng/mL cholera toxin, 10 ng/mL epidermal growth factor and 1% antibiotic–antimycotic. The limbal epithelial cells were then trypsinized in TrypLE Express for 5 min and scraped. The resulting cell suspension was pelleted by centrifugation at 400× *g* for 5 min and resuspended in sGMedium.

For the culture of human limbal epithelial cells, growth-arrested 3T3-J2 fibroblasts were suspended in DMEM GlutaMAX™ with 10% fetal bovine serum and plated at a density of 4.6 × 10^4^ cells/cm^2^ in a 100-mm tissue culture dish (Corning, Glendale, AZ, USA). On the following day, limbal epithelial cells were harvested from corneoscleral tissues, as described above, and seeded in 100-mm tissue culture dishes containing growth-arrested 3T3-J2 fibroblasts in sGMedium. The medium was changed on the third day and every other day thereafter. After 7 days, the 3T3-J2 feeder cells were detached using Versene (Thermo Fisher Scientific) for approximately 1 min, followed by a wash in Dulbecco’s modified phosphate-buffered saline, and a 10-min incubation with TrypLE Express to detach the epithelial cells. These cells were pelleted by centrifugation and frozen in DMEM GlutaMAX™ supplemented with 20% fetal bovine serum and 10% DMSO. For the routine passage of cells in clonogenic assays, frozen human limbal epithelial cells from the cell bank were plated at a density of 1-2 × 10^3^ cells/cm^2^ in the presence of 3T3-J2 fibroblasts. For the differentiation assays, cells were plated and passaged at a density of 5 × 10^4^ cells/cm^2^ in the absence of fibroblasts.

### 2.3. Isolation of Individual Clones

Individual clones were isolated by culturing human limbal epithelial cells (passage 2 to 4) at a density of 15 cells/cm^2^ in the presence of growth-arrested 3T3-J2 fibroblasts. After 7 days, the 3T3-J2 feeder layer was detached with Versene, and a Scienceware cloning cylinder (MilliporeSigma) was set over individual clones before trypsinization with TrypLE Express. Once detached, the cell suspension was pelleted by centrifugation, resuspended in sGMedium and distributed as follows: one-fourth of each individual clone was seeded into a 100-mm tissue culture dish (indicator dish) containing growth-arrested 3T3-J2 fibroblasts for clonal classification. The remaining cells were centrifuged and lysed in 5 µL of buffer RLT (Qiagen, Valencia, CA, USA) for the transcriptional assay. The cell lysates were stored at −80 °C until further processing.

### 2.4. Clonal Classification

Indicator dishes were grown for 12 days in sGMedium for the classification of the clonal type [14,15]. At that point, the 3T3-J2 fibroblasts were detached from the culture and the colonies were fixed in 4% paraformaldehyde (Alfa Aesar, Tewksbury, MA, USA) for 15 min at room temperature. Dishes were washed with Dulbecco’s modified phosphate-buffered saline lacking calcium and magnesium and stained with 2% Rhodamine B (Sigma-Aldrich, St. Louis, MO, USA) or 0.2% crystal violet (Sigma-Aldrich). After washing, the cell cultures were imaged using a G:Box (Syngene, Frederick, MD, USA) and analyzed with ImageJ software (available in the public domain at http://rsbweb.nih.gov/ij/ (accessed on 12 August 2019)). The clonal type was determined by the percentage of aborted colonies formed by the progeny of the clone in the indicator dish. When all colonies formed were terminal, the clone was classified as abortive. Clones leading to half or fewer terminal colonies were referred to as clonogenic.

### 2.5. RNA Isolation and cDNA Synthesis

Total RNA was isolated from frozen individual clones using the RNeasy Micro Kit (Qiagen) following the manufacturer’s instructions. Residual genomic DNA in the RNA preparation was eliminated by on-column DNase I digestion (Qiagen). For the PCR array, RNA concentration and purity were assessed using the Agilent RNA 6000 Pico Kit (Agilent, Santa Clara, CA, USA). Analyses were performed with total RNA isolated from three donor tissues. The abortive (8–9 clones) or clonogenic (3 clones) cells produced by each tissue were pooled and the RNA was amplified using the Ovation RNA-Seq System V2 (Tecan Genomics Inc., Redwood City, CA, USA) according to the manufacturer’s protocol. Then, the cDNA was purified using a MinElute Reaction Cleanup Kit (Qiagen), quantified on a NanoDrop 2000 spectrophotometer, and the purity was evaluated using the Agilent RNA 6000 Nano Kit. For qPCR, total RNA was transcribed using the iScript^TM^ cDNA Synthesis Kit (Bio-Rad, Hercules, CA, USA).

### 2.6. Human Glycosylation PCR Array

The analysis of the genes encoding glycosylation enzymes was carried out using a human glycosylation PCR array (RT2 Profiler^TM^ PCR array, Qiagen) according to the manufacturer’s instructions. The ΔΔC_T_ and 2^−ΔCT^ methods were used for the relative quantitation of the number of transcripts. Data were analyzed using the web-based PCR array data analysis software (SABiosciences, Available online: http://www.sabiosciences.com/ (accessed on 12 April 2022)).

### 2.7. qPCR

The qPCR was performed using the SsoAdvanced™ Universal SYBR^®^ Green Supermix (Bio-Rad). Primer sequences for *MUC16* (Unique Assay ID qHsaCID0018430), *GCNT4* (Unique Assay ID qHsaCED0047215), *FUCA2* (Unique Assay ID qHsaCID0007217) and *GAPDH* (Unique Assay ID qHsaCED0038674) mRNA were obtained from Bio-Rad. Gene expression was measured in a Mastercycler ep realplex thermal cycler (Eppendorf, Hauppauge, NY, USA) with the following parameters: 2 min at 95 °C, followed by 40 cycles of 5 s at 95 °C and 30 s at 60 °C. Fold changes were calculated using the comparative ΔΔC_T_ method by normalizing to *GAPDH*.

### 2.8. Statistical Analyses

All statistical analyses were performed using Prism 9 (GraphPad Software, San Diego, CA, USA) for Mac OS X.

## 3. Results and Discussion

### 3.1. Glycogene Expression Profile in Human Limbal Epithelial Cells

**Figure 1:**
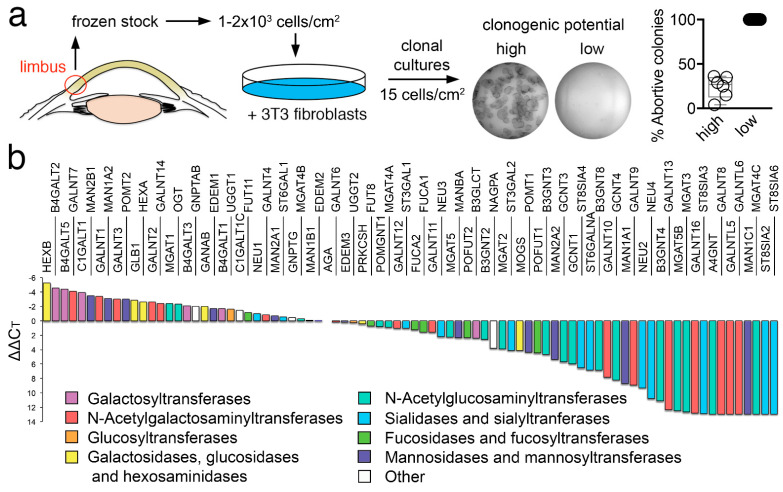
Glycogene expression profile in human limbal epithelial cells. (**a**) Schematic diagram showing the strategy to isolate individual clones with different clonogenic potential. (**b**) Assessment of the relative transcript abundance of genes encoding glycosylation enzymes in clonogenic cells using a pathway-focused PCR array. The expression of genes was normalized using the comparative ∆∆C_T_ method.

We first produced a frozen stock of human limbal epithelial cells to allow the expansion and isolation of individual clones. Toward this goal, a cell bank was created with limbal epithelial cells extracted from human corneoscleral tissues. These cells were grown in the presence of fibroblasts and individual populations isolated by circumscribing well-defined foci with cloning cylinders. The clonogenic potential of these focal populations was assessed by culturing them into indicator dishes, followed by direct visualization of the total number of terminal colonies. Clones leading exclusively to the terminal colonies were referred to as abortive, also known as paraclone, while populations in which only half or less of the clones were terminal were referred to as clonogenic (Figure 1a). One limitation of this classification is that the clonogenic cultures comprise a mixture of holoclones and meroclones. Holoclones have been defined as populations of cells capable of extensive proliferation and self-renewal, whereas meroclones contain a mixture of cells of different growth potential, representing a transitional stage between the holoclone and the paraclone [14,15]. It is important to note, however, that the number of cells that can be obtained from human corneoscleral tissues is limited. Therefore, our classification allowed us to generate sufficient material to perform transcriptional analyses in two groups of cells with different clonogenic potentials.

Next, we examined the transcriptional levels of known glycosylation enzymes in the two cell populations using a pathway-focused PCR array. A direct comparison of the relative levels of expression in the clonogenic population revealed a high content of transcripts regulating galactose and mannose metabolic pathways (Figure 1b). Two of the most highly expressed glycogenes were *B4GALT5* and *C1GALT1*. B4GALT5 is a glycosyltransferase responsible for the addition of galactose to N-glycans on proteins and carbohydrate moieties of glycolipids, and whose expression has been associated with the maintenance of stemness in breast cancer stem cells by inducing Frizzled-1 glycosylation and by constantly activating the canonical Wnt signaling pathway [16]. C1GALT1, on the other hand, catalyzes the early addition of galactose to mucin-type O-glycans, resulting in the synthesis of the T antigen (Galβ1-3GalNAc) and has been shown to be prominently expressed in embryonic stem cells, where it maintains pluripotency by modulating Wnt receptor endocytosis [17]. We also found that limbal epithelial cells with clonogenic potential were enriched in *MAN1A2* and *MAN2B1*, two mannosidases involved in the maturation of N-glycans and their degradation in the lysosome, respectively.

### 3.2. Abortive Clones Contain High Levels of GCNT4 and FUCA2

**Figure 2:**
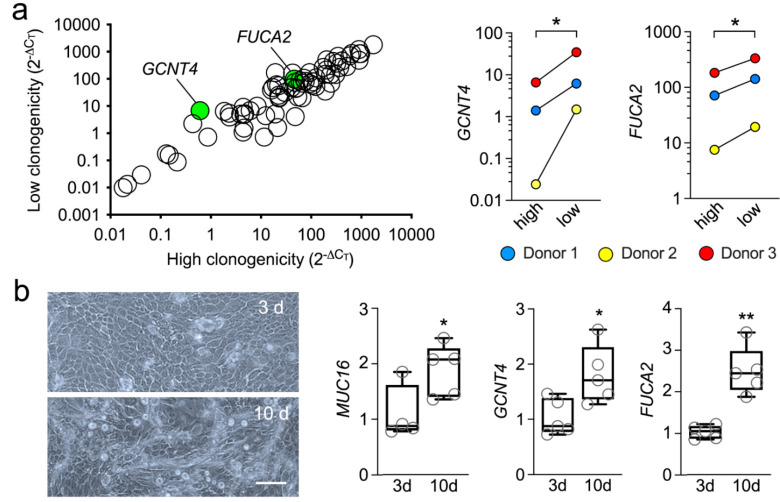
Abortive clones contain high levels of *GCNT4* and *FUCA2*. (**a**) Scatterplot comparing the expression of glycogenes in cells with different clonogenic potential (n = 3 independent donors). The green dots indicate statistically significant upregulation of the glycogene. The corresponding quantitative graphs are shown to the right. (**b**) Microscopic appearance of primary human limbal epithelial cells cultured at confluency for 3 or 10 days without supporting fibroblasts. By qPCR, *MUC16*, *GCNT4* and *FUCA2* mRNA were significantly upregulated after culture for 10 days (n = 4-5 independent experiments). The data in (**a**) are presented as individual paired values. The box-and-whisker plots in (**b**) show the 25 and 75 percentiles (boxes), the median, and the minimum and maximum data values (whiskers). Significance in (**a**) was determined using a multiple paired t-test and in (**b**) using the unpaired t-test or Mann–Whitney test for nonparametric data. Scale bar: 100 μm. *, *p* < 0.05; **, *p* < 0.01.

Paraclone or abortive clones contain, primarily, cells with a short replicative lifespan committed to differentiation [14]. The transcriptional analysis of this population revealed multiple glycogenes that were up- or down-regulated by at least two-fold, compared to the clonogenic cells (Supplementary Table S1). However, we found that most of these changes were not statistically significant, although it could be argued that there is intrinsic variability across the human population in terms of gene expression and that the sample size in our study was relatively small. The only two genes that were significantly altered in the abortive clones were *GCNT4* and *FUCA2* (Figure 2a). Furthermore, that *GCNT4* and *FUCA2* were significantly upregulated in clones committed to differentiation was further confirmed using an in vitro model, where primary human limbal epithelial cells were cultured at confluency for 10 days without supporting fibroblasts (Figure 2b). In these experiments, the expression of MUC16, a transmembrane mucin known to localize to the tips of microplicae on the apical cells of the native and cultured corneal epithelium, was used as a marker of terminal differentiation [18].

GCNT4 is a glycosyltransferase that mediates O-glycan core branching, an important step in the processing of mucin-type glycoproteins. The observation of increased expression of an enzyme involved in the maturation of mucin-type O-glycans in abortive clones compared to clonogenic cells was not entirely surprising, since increased core diversity characterizes the O-glycans present in differentiated cell types, including human corneal epithelial cells [19,20]. On the other hand, detecting increased *FUCA2* levels in the abortive clones was somehow unexpected, since differentiated human corneal epithelial cells contain extensive fucosylated structures [21,22]. FUCA2 is an exoglycosidase that catalyzes the removal of terminal fucose from glycoproteins present in the glycocalyx and extracellular matrix. One possible explanation for the presence of the extensive fucosylated structures in differentiated cells, however, is the presence of other pathways of fucose metabolism that could overcome the catalytic functions of FUCA2. Interestingly, supplementing breast cancer cells with exogenous FUCA2 protein has been shown to reinforce their colony-forming ability, suggesting that a reduced fucose environment supports the growth potential of these cells [23]. We believe that future experiments aimed at further defining the glycome of the human cornea should provide additional insight into the involvement of specific carbohydrate pathways in maintaining the limbal stem cell phenotype.

## 4. Conclusions

A number of studies have provided insight into the glycome of ocular surface epithelial cells, with a clear focus on pathological processes, such as those observed in dry eye, wound healing, and ocular rosacea [24]. However, there is only a superficial understanding of whether limbal stem cells have a unique glycome that differentiates them from mature cells and, if so, what factors contribute to regulating the glycosylation of the limbal stem cell niche. Early studies in rabbits evidenced the lack of fucosylated Le^x^ in the limbal region of the cornea, leading to speculation that this determinant contributes to maintaining an undifferentiated state in stem cells, whereas its induction would initiate terminal differentiation [25]. The more recent investigation into the composition of the extracellular matrix of the limbal niche in mice has shown that it is composed of specialized hyaluronan, a high molecular weight glycosaminoglycan, that differs from that present in the rest of the corneal epithelium [26]. These and other studies are leading to the use of hyaluronan-based hydrogel scaffolds for the treatment of limbal stem cell deficiency [27]. Our data provide a further understanding of the composition of the glycome in human limbal epithelial cells in relation to their clonogenic potential and constitute the basis for subsequent studies to determine the functional relevance of this important biological process.

## Data Availability

The data presented in this study are available in the article.

## Supplementary Materials

The following supporting information can be downloaded at: https://www.mdpi.com/article/10.3390/cells11091575/s1, Supplementary Table S1: Relative expression (ΔC_T_) of genes encoding glycosylation enzymes in clonogenic human limbal epithelial cells.
