# Supplementary material for: Glycogene Expression Profile of Human Limbal Epithelial Cells with Distinct Clonogenic Potential
Source: Cells. 2022 May 7;11(9):1575. doi: 10.3390/cells11091575 (PMC9102009; doi:10.3390/cells11091575)
Supplement: Supplementary file 1 [file cells-11-01575-s001.zip › cells-1703942-supplementary.pdf]

Supplemental Table S1. Relative expression (ΔC_T) of genes encoding glycosylation enzymes in clonogenic human limbal epithelial cells. High positive ΔC_T values reflect low amplification efficiency. Values in bold indicate that gene expression was significantly higher in abortive colonies compared to clonogenic ($2^{-\Delta C_T}$).

Gene Symbol	RefSeq*	Magnitude of Expression (ΔC_T)	Abortive Fold Change ($2^{-\Delta C_T}$)
N-Acetylgalactosaminyltransferases			
GALNT1	NM_020474	0.63	1.32
GALNT2	NM_004481	1.63	1.16
GALNT3	NM_004482	0.98	0.56
GALNT4	NM_003774	3.72	1.06
GALNT6	NM_007210	4.20	0.40
GALNT7	NM_017423	0.16	1.35
GALNT9	NM_021808	12.72	1.05
GALNT10	NM_198321	11.16	5.15
GALNT11	NM_022087	5.45	0.87
GALNT12	NM_024642	5.07	1.06
GALNT14	NM_024572	2.03	0.59
GALNT16	NM_020692	15.49	0.60
N-Acetylglucosaminyltransferases			
B3GNT2	NM_006577	5.87	2.79
B3GNT3	NM_014256	7.72	1.10
B3GNT4	NM_030765	12.19	0.41
B3GNT8	NM_198540	10.16	0.83
GCNT1	NM_001490	8.75	2.35
GCNT3	NM_004751	7.82	2.04
GCNT4	**NM_016591**	**10.68**	**11.26**
MGAT1	NM_002406	2.38	0.19
MGAT2	NM_002408	7.99	1.29
MGAT4A	NM_012214	5.54	0.08
MGAT4B	NM_014275	3.80	1.48
MGAT5	NM_002410	5.61	2.98
MGAT5B	NM_144677	15.77	0.55
OGT	NM_181673	2.02	0.28
POMGNT1	NM_017739	4.61	1.18
Galactosyltransferases			
B4GALT1	NM_001497	2.53	1.52
B4GALT2	NM_003780	0.11	0.92
B4GALT3	NM_003779	1.85	1.57

B4GALT5	NM_004776	0.04	0.87
C1GALT1	NM_020156	0.15	0.54
Glucosyltransferases			
UGGT1	NM_020120	2.29	2.48
UGGT2	NM_020121	4.34	0.90
Mannosidases			
EDEM1	NM_014674	3.00	0.87
EDEM2	NM_018217	3.91	1.05
EDEM3	NM_025191	4.30	0.92
MAN1A1	NM_005907	6.89	1.18
MAN1A2	NM_006699	1.29	1.11
MAN1B1	NM_016219	4.07	1.44
MAN1C1	NM_020379	3.20	0.72
MAN2A1	NM_002372	9.07	3.43
MAN2B1	NM_000528	0.74	1.19
MANBA	NM_005908	5.69	8.79
Mannosyltransferases			
POMT1	NM_007171	7.76	0.34
POMT2	NM_013382	1.54	1.98
Galactosidases, glucosidases and hexosaminidases			
GANAB	NM_198334	2.31	1.04
GLB1	NM_000404	1.70	0.47
HEXA	NM_000520	1.42	0.71
HEXB	NM_000521	-0.80	1.04
MOGS	NM_006302	7.77	1.07
PRKCSH	NM_002743	4.39	1.58
Fucosidases and fucosyltransferases			
FUCA1	NM_000147	5.47	1.12
FUCA2	**NM_032020**	**4.44**	**2.11**
FUT8	NM_178157	4.55	3.83
FUT11	NM_173540	3.40	0.70
POFUT1	NM_172236	8.73	1.68
POFUT2	NM_133635	6.42	0.06
Sialidases			
NEU1	NM_000434	3.24	0.55
NEU2	NM_005383	12.90	1.35
NEU3	NM_006656	5.49	2.50

NEU4	NM_080741	14.57	0.71
Sialyltransferases			
ST3GAL1	NM_173344	5.44	0.93
ST3GAL2	NM_006927	7.83	0.25
ST6GAL1	NM_003032	4.38	0.09
ST6GALNAC1	NM_018414	5.95	2.43
ST8SIA4	NM_175052	5.70	0.27
Mannose-6-phosphate synthesis and catabolism			
GNPTAB	NM_024312	2.39	0.77
GNPTG	NM_032520	3.43	1.83
NAGPA	NM_016256	7.36	1.12
Other glycosylation genes			
AGA	NM_000027	3.92	0.30
C1GALT1C1	NM_152692	3.02	0.43

*Reference sequence database at NCBI (https://www.ncbi.nlm.nih.gov/refseq/)
